# Influence of bioactive metal fillers on microstructural homogeneity of PA12 composites produced by polymer Laser Sintering

**DOI:** 10.1007/s43452-022-00442-4

**Published:** 2022-05-05

**Authors:** Piotr Gruber, Grzegorz Ziółkowski, Michał Olejarczyk, Emilia Grochowska, Viktoria Hoppe, Patrycja Szymczyk-Ziółkowska, Tomasz Kurzynowski

**Affiliations:** Department of Laser Technologies, Automation and Production Management, Faculty of Mechanical Engineering, Centre for Advanced Manufacturing Technologies, Wrocław University of Science and Technology, Wrocław, Poland

**Keywords:** Polymer laser sintering (pLS), Polyamide 12 (PA12), Composites, Antibacterial, Computed tomography (CT)

## Abstract

In this paper, polyamide 12 (PA12) blends with three types of metallic fillers are tested, which differ in the type of material and its’ morphology. Low content mixtures are taken into consideration (0.5, 1.0, 2.0, 5.0 wt%), since a low impact on mechanical properties along with obtaining antibacterial properties are desired. The investigation focuses on filler distribution as well as the influence on microstructural homogeneity of the base material after processing with polymer Laser Sintering. Moreover, the influence of the filler content on the mechanical properties and fracture behaviour were examined. Processability PA12 with bioactive metal fillers was confirmed, and no significant changes in ductile behaviour of PA 12 were observed. An in-depth analysis of the effect of the filler on microstructural homogeneity was conducted.

**Graphical abstract:**

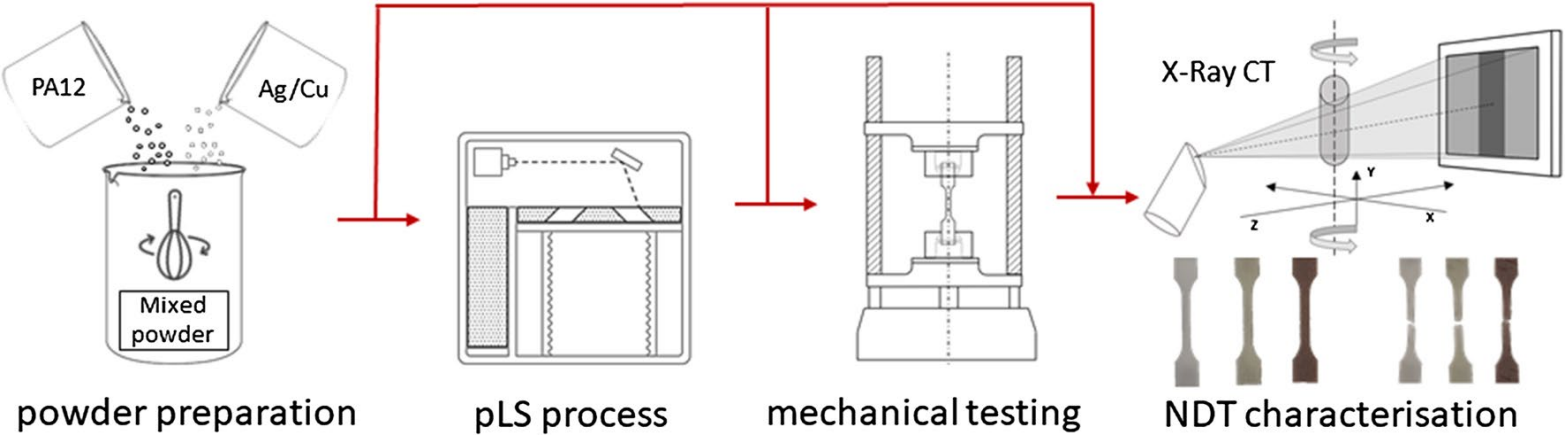

## Introduction

Polymer laser sintering (or pLS) is one of the most representative polymer-based Additive Manufacturing (AM) technologies assigned to the laser-based powder bed fusion of polymers (LB-PBF/P) [1]. pLS uses a laser source to selectively fuse polymer powder particles based on digital CAD 3D data. Besides the laser radiation, a typical pLS machine preheats the raw material to near-melting temperature using a heater, which in most cases are infrared lamps or resistance heaters. Polymer powder is spread across the build area using a recoater or a roller with a counter-rotating movement. The recursive process of powder application, preheating, fusing and lowering the work platform by a given layer thickness is repeated until the desired three-dimensional part is built.

Due to the COVID-19 pandemic, the demand for antibacterial polymer materials significantly increased, especially visible within the market for a related AM technology—Fused Filament Fabrication materials [2]. More than few filament producers decided to expand their product portfolio to antibacterial filaments (PLA, TPU, or PET-G), which have a bioactive filler, such as copper. The market dedicated to LB-PBF/P includes some filled powders with fillers such as glass beads, carbon fiber, or aluminium. Each material’s goal is to improve functional properties such as temperature resistance, stiffness, strength, surface finish, or even ease of machinability. On the market, a material with bioactive fillers such as copper or silver is missing, despite the fact that there is a visible growth in the research of polymer powder composites [3], not only with metallic fillers such as silver [4] or copper [5] but also with ceramic [6], or organic including carbon [7] and wood [8]. Active ingredients are also reported, filling the polymer matrix with drugs such as progesterone [9] or indomethacin [10]. Scientists are already working on the production of polymer and metal-based composites that can be produced by mechanically mixing a polymer with metal nanoparticles, based on technologies other than AM [11].

Research into AM of antimicrobial materials is a small but rapidly developing field. The development of materials that will not transmit additional microorganisms during the COVID-19 pandemic is particularly important due to the high involvement of AM technology in the implementation of new products for use in hospitals. Antimicrobial polymers offer increased efficacy among available antimicrobial agents with the potential to minimize health and environmental problems thanks to the use of innovative technological and product solutions, which are especially important in times of a pandemic. The development of a polymer exhibiting effective antibacterial properties using AM appears to be increasingly important due to the wide use of polymers in prototyping critical medical devices. Palza [12] suggests that the addition of copper nanoparticles to polymers and their antimicrobial properties are promising applications for their implementation in the development of medical devices related to bacterial growth.

In the context of the COVID-19 pandemic, the choice of copper and silver as fillers was dictated by the strong antiviral effect of copper [13] and antimicrobial properties of silver [14]. Many surfaces can be contaminated with people’s hands: viruses and bacteria deposited on objects can be dispersed by aerosols or left in contaminated body fluids, leading to the rapid transmission of pathogens. The use of surfaces with biocidal properties plays an important role in combating many other infections because their application prevents the multiplication of microorganisms. Antimicrobial material processed with additive manufacturing would allow the manufacturing of end-use parts, which can be implemented in sectors such as healthcare, public transportation or public utility buildings.

The crucial factor and the aim of this study is the selection of such parameters of the batch material and process parameters to obtain a homogeneous structure with an even distribution of particles throughout the volume of the produced element. Such a solution is important from the point of view of antibacterial properties and uniformity throughout the volume of mechanical properties of objects manufactured using pLS technology. In the literature there is no complex study considering the influence of bioactive metallic fillers on material microstructural homogeneity after pLS, particularly for low content blends. Balzereit et al. consider the use of PA12 (PA2210FR) mixtures with 5, 10 and 20 wt% copper for use in electronic circuit carriers [15]. Research shows increased porosity for 5 wt% copper content, which is non-favourable in the case of maintaining good mechanical properties of laser sintered PA12. Wolf et al. report a decrease in the ductile character of PA12 with 10 wt% of copper powder [16]. Lantz et al. draw attention to changes in temperature conductivity and heat capacity due to the use of metallic fillers. Differences in thermal properties which ultimately will lead to process parameter optimization, which has to be set according to the filler content [5]. Turner et al. report using silver glass (B65003) with 1 wt% blend of PA12 (PA2200) for achieving antimicrobial properties, and shows only slight changes in mechanical properties [4].

In this paper, PA12 blends with three types of metallic fillers are tested, which differ in the type of filler material as well as morphology. The investigation focuses on mechanical properties, filler distribution as well as influence on microstructural homogeneity of base material after processing with pLS. No in-depth analysis on the influence of type and content is found in the existing literature. Low content blends are considered (0.5, 1.0, 2.0, 5 wt%) due to the literature reporting undesirable changes in mechanical properties, especially when considering PA12 ductile character that is desirable for functional parts.

## Materials and methods

### Powder mixture preparation

For the experiment, physical powder mixtures were made using the base material, virgin (mixing ratio 100/0) PA12 polymer (PA2201, EOS GmbH, Krailling, Germany), and the fillers, two types of copper powder [spherical by ECKA Granulate Velden GmbH, Velden, Germany—referred as Cu(S) and dendritic by WARCHEM Sp. z o.o., Warsaw, Poland referred as Cu(D)] and one silver powder (KGHM Polska Miedź S.A., Lubin, Poland, referred as Ag). The mixture was physically mixed with the matrix material in a weight ratio of 0.5%, 1.0%, 2.0% and 5.0%, respectively (Table 1). The mixing procedure was carried out in a sealed half-filled container, rotated 45° from the axis around which rotation was performed at a speed of 20 rpm. Subsequently, the powder characteristics were tested for the base powder and the mixtures.

**Table 1:** Characteristics and weight composition of powder mixtures

Weight	PA2201 (ref.)	PA2201 + Ag	PA2201 + Cu(S)	PA2201 + Cu(D)
%	0	0.5	0.5	0.5
		1.0	1.0	1.0
		2.0	2.0	2.0
		5.0	5.0	5.0

### Batch material and filler characterization

The shape of matrix polymer PA2201 and metal fillers were analyzed by scanning electron microscopy (SEM) using an EVO MA25 device (Zeiss, Oberkochen, Germany) with accelerating voltage of 20 kV. To achieve high-resolution imaging of polymer powders to increase conductivity, sputtering a thin layer of gold was performed using a Q150R ES device (Quorum Technologies Ltd, Lewes, United Kingdom). Quantitative evaluation of the metal particle shape was performed with the use of X-ray computed tomography (XCT) (see chapter 3.5). The particle size distribution (PSD) was determined with the use of laser diffraction technology. The system consisting of HELOS/BR (laser diffraction sensor) and RODOS (dry dispersion unit) and VIBRI/L (feeder) (Sympatec GmbH, Clausthal-Zellerfeld, Germany) were used for this purpose. The PSD test was carried out according to the ISO 13220-1 standard. The dispersing method was set at 2 bar and used a vibration material feeder. The feed rate was set to 100% with gap width at 3.0 mm for polymer powder, and 90% and 2.0 mm for metallic powders.

### Manufacturing process

**Fig. 1:**
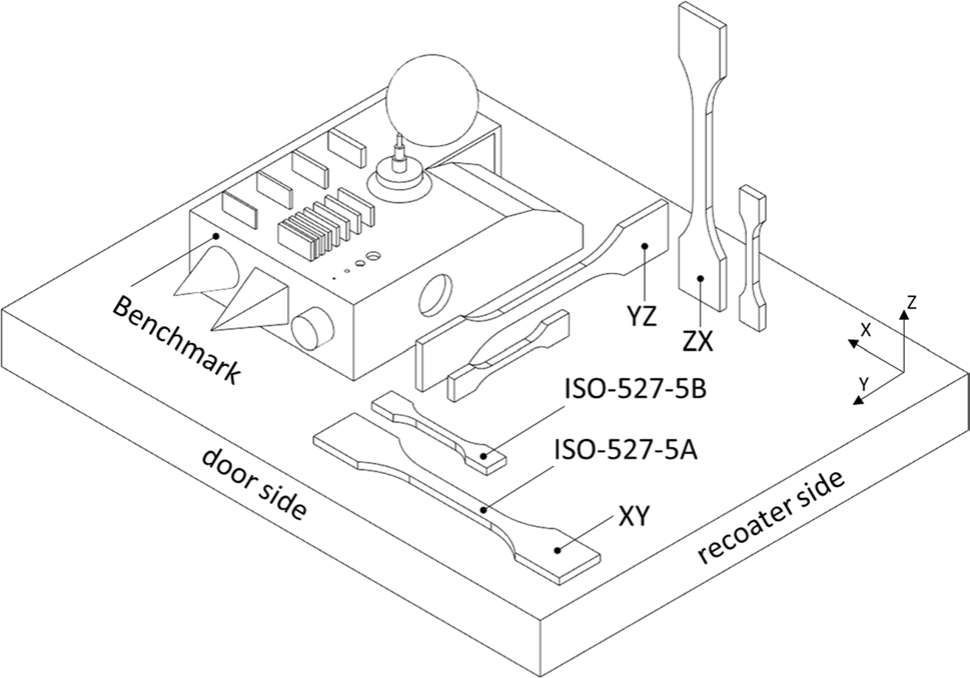
Benchmark geometry and test samples prepared according to ISO-527-5A and ISO-527-5B with its build orientations

The manufacturing process was carried out using an EOS Formiga P110 (EOS GmbH, Krailling, Germany) with a standard set of parameters dedicated to PA2201 powder (scanning style EOS) and a layer thickness of 100 µm. Processing temperatures were determined according to the procedure recommended by the manufacturer and set at 171 °C (on print surface) and 151 °C (in the removal chamber). Each mixture was processed separately, while layout and the orientation of samples were not changed. Besides the test samples a benchmark geometry including basic geometrical shapes was built. The benchmark model (50 mm × 90 mm × 37 mm), shown in Fig. 1, was used to verify the ability to process mixture materials and manufacture models without errors.

### Mechanical testing

The static tensile test was carried out using an Instron 3384 testing machine with a maximum load of 10 kN equipped with a video extensometer AVE 2663-821 (Instron, Norwood, MA, United States of America) and Multitest-I (Mecmesin Ltd, West Sussex, United Kingdom) test frame with a force sensor with a maximum load of 1 kN. Both tests were carried out using a speed of 10 mm/min. As part of the experiment, 13 different series of dog bone-shaped samples were prepared. The series differ in the weight percentage of copper or silver. The samples in the series were manufactured in three different build orientations: *XY*, *YZ*, *ZX* according to ISO/ASTM52921:2013, defined as *XYZ*, *YZX*, *ZXY*. Two types of tensile specimens were prepared according to ISO-527-5A (tested on Instron 3384) and ISO-527-5B (tested on Mecmesin Multitest-I) standards. Smaller tensile specimens (ISO-527-5B) were prepared specifically for XCT examinations, in which the size of the sample affects the test resolution. A total of 390 tensile specimens were produced, 5 for each type, series and build orientation.

### Fractography

Fractography analysis was conducted using microscopic method. The fractured surface of tensile specimens (ISO-527-5A) of 13 different series samples with built orientation of *XYZ* and *ZXY* were examined. Fractography were transverse planes of vertical and horizonal orientations with digital microscope VHX-6000 (Keyence, Osaka, Japan). Image acquisitions was made with lens with × 30 magnification.

### NDT characterisation with X-ray computed tomography (XCT)

The XCT system used was the phoenix v|tome|x m 300/180 (GE Sensing & Inspection Technologies GmbH, Wunstorf, Germany), which is equipped with two x-ray tubes with a cone beam: a transmission tube (nanofocus, max. voltage of 180 kV) and a directional tube (microfocus, max. voltage of 300 kV); and a matrix detector with a resolution of 2000 × 2000 pixels and a pixel size of 200 µm. The phoenix datos|× 2.7.2 software allowed for a three-dimensional reconstruction of the data from the two-dimensional projections collected during the XCT scanning.

**Fig. 2:**
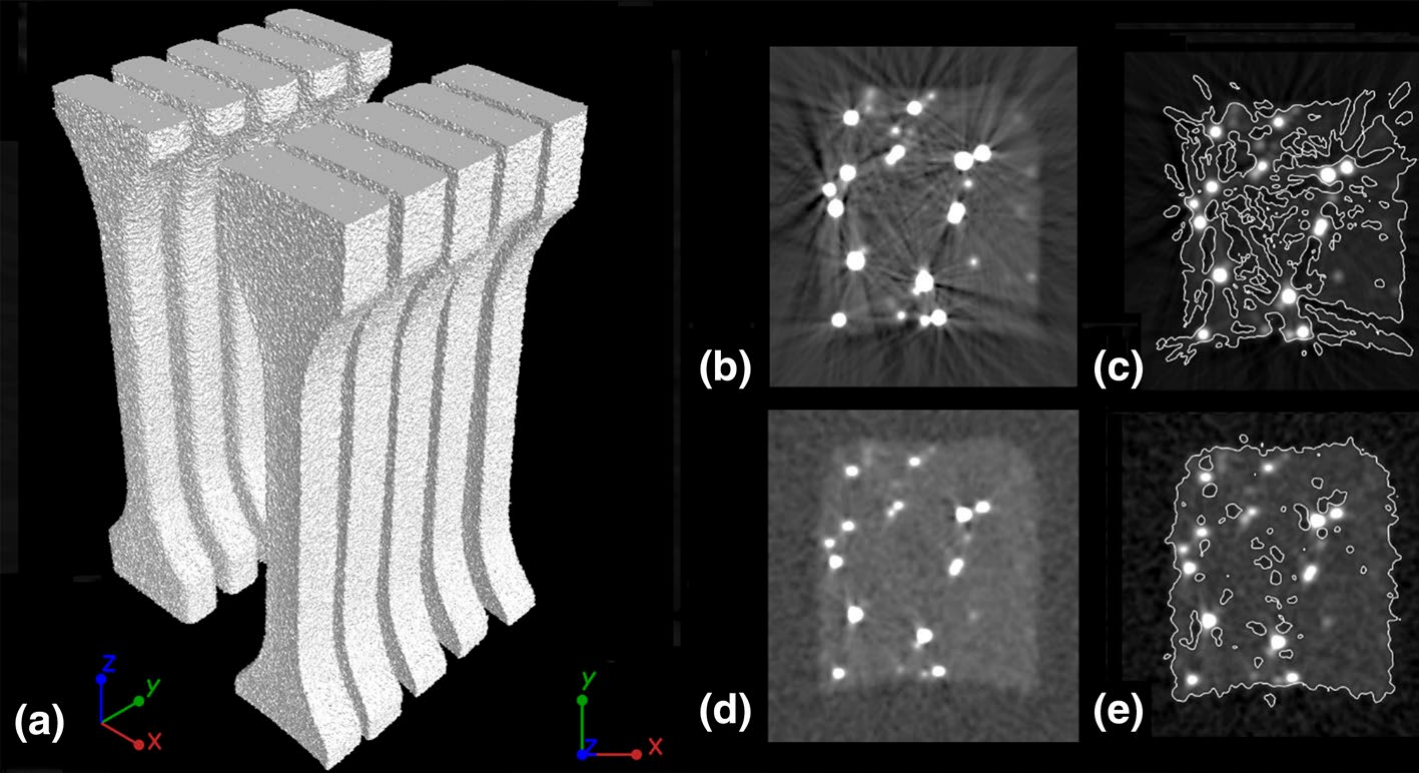
Result of 3D reconstruction of ISO-527-5B samples after scanning, **b** 2D cross-section through the sample before measurement parameter optimization, **c** 2D cross-section through the sample showing the detected edge before parameter optimization, **d** 2D cross-section through the sample after parameter optimization, **e** 2D cross-section through the sample showing the detected edge after parameter optimization

The test samples (ISO-527-5B) were scanned with the use a microfocus X-ray tube with a resolution of 20 µm, which allowed for scanning 10 samples at the same time (Fig. 2a), thanks to which a total of 130 samples were tested. Particular attention was paid to the selection of measurement parameters, which were as follows: X-ray tube voltage 230 kV, current 70 µA, exposure time 250 ms, and the number of projections was 1800. To eliminate the adverse effects of artefacts, i.e. various irregularities (X-ray scattering) recorded in the polymer matrix around metal fillers (Fig. 2b, c), a physical filter in the form of a copper plate with a high thickness of 2.5 mm was used. This allowed for a significant reduction of measurement artefacts (Fig. 2d) and the determining the distinction of the boundary between the polymer matrix and the pores inside the samples (Fig. 2e).

**Fig. 3:**
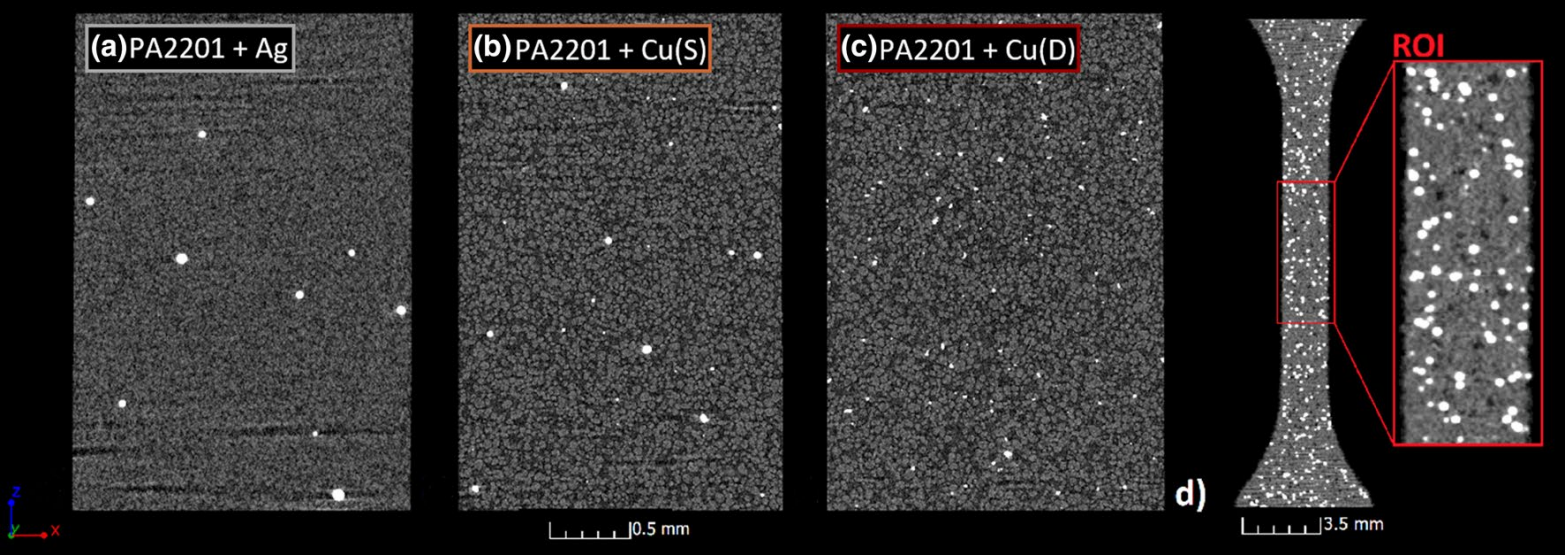
Powder mixtures with **a** Ag, **b** Cu(S), **c** Cu(D) and PA2201 as a base material; **d** ROI used to analyse with high resolution distribution of metal particles in the sample

Due to the high scanning resolution necessary for accurate evaluation of the powder particles, a nanofocus X-ray tube was used to analyse the shape of the fillers (Cu and Ag), and to analyse the distribution of metal particles in the sample. In the case of the particle shape, special samples were prepared by mixing Cu and Ag powder grains with PA2201 powder. After mixing, 20 µl powder samples without compacting were reconstructed (Fig. 3a–c). In the case of analysis of the distribution of the powder grains after the pLS process, only the ROI (Region of Interest) with a height of 5 mm in the middle of the tested samples was reconstructed (Fig. 3d).

This made it possible to obtain, in both cases, measurements with a voxel size of 2.9 µm. The remaining scanning parameters were as follows: X-ray tube voltage 120 kV, current 100 µA, exposure time 250 ms and the number of projections equal to 1600. In this case, filtration was applied, in the form of 0.3 mm of copper plate. The analysis of the XCT data was performed with the use of VG Studio MAX 3.3 (Volume Graphics GmbH, Heidelberg, Germany).

## Results and discussion

### Powder mixture preparation

The prepared powder mixtures were characterized by a uniform colour, changing from white (PA2201 without fillers) to a light shade of yellow (Ag) or red (Cu) with an increase in the filler content. Non-homogeneous filler agglomerations or compacted powder particles were not observed. The mixing time used was initially considered as suitable for ensuring a homogeneous powder blend.

### Batch material and filler characterization

**Fig. 4:**
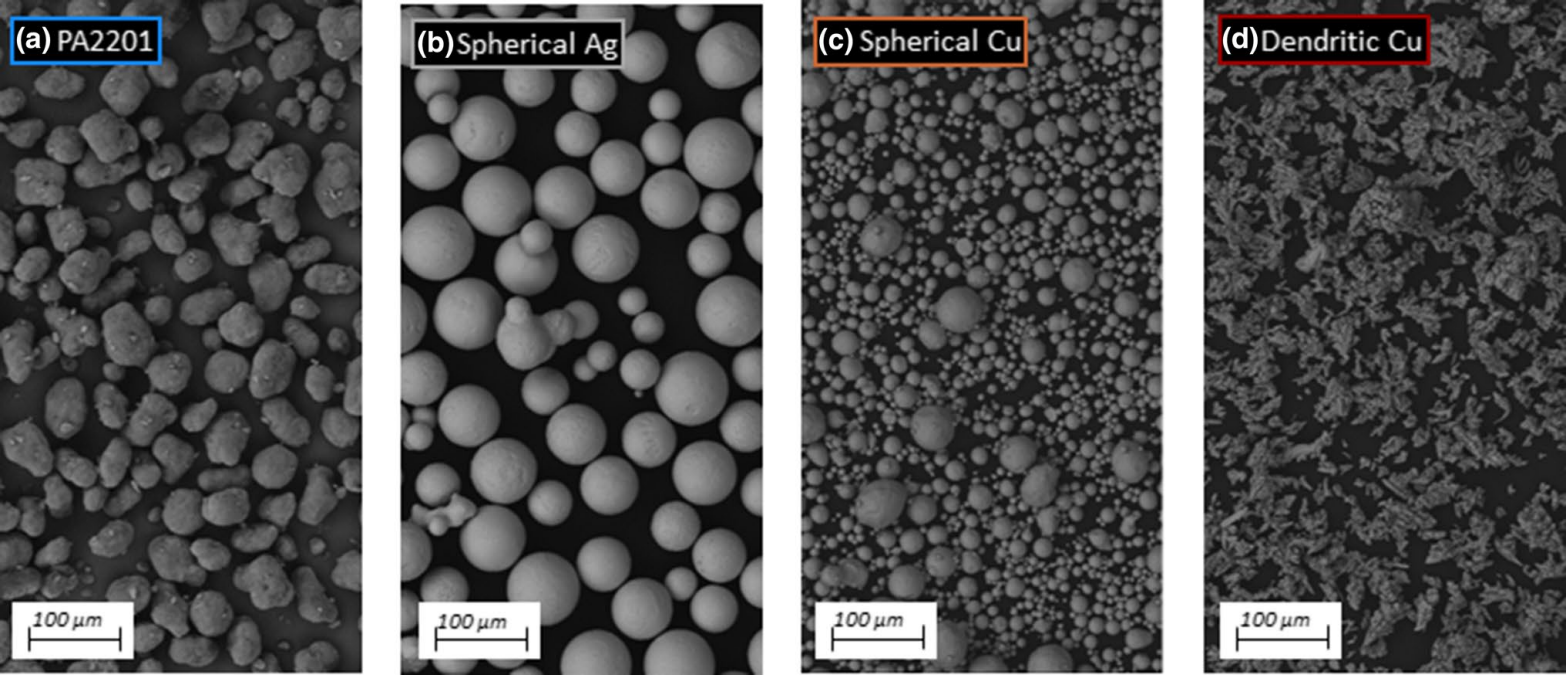
SEM images of polymer (base) and matallic powders (fillers): **a** PA2201; **b** spherical Ag (Ag); **c** spherical Cu (Cu(S)); Dendtiric Cu (Cu(D))

The powder particles comprising the filler were characterized using SEM imaging, presented in Fig. 4. The polymer PA2201 powder, constituting the matrix of the mixture, is characterized by a near spherical or nearly elliptical shape, which is referred to in the literature as a potato shape. The powders used for this experiment were silver powder and copper powders with two different morphologies. Silver powder is characterized by a spherical shape of particles with low size differentiation, which was confirmed by PSD tests. In the images of the copper powders, significant differences in the shape of the particles can be seen. One of them is a dendritic powder, while the other is a pseudo-spherical powder.

**Fig. 5:**
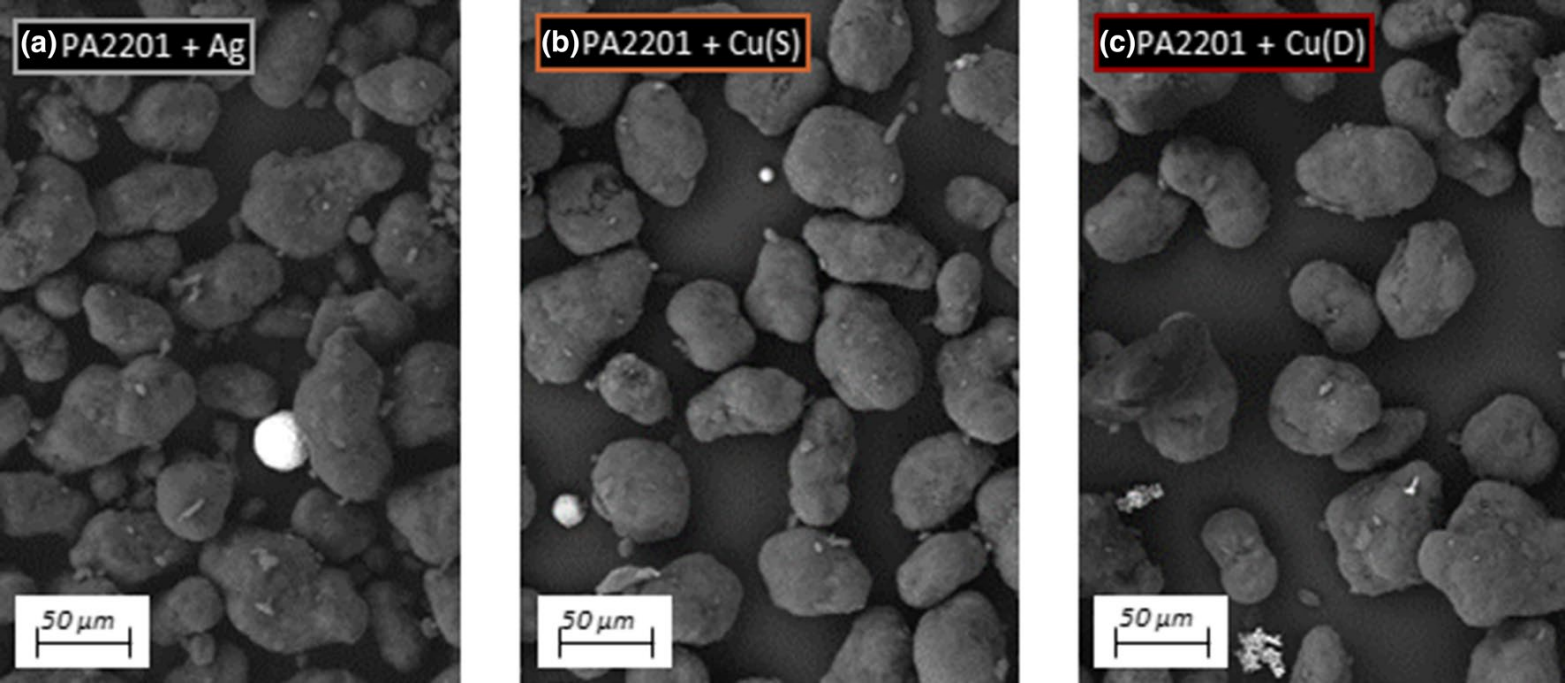
SEM images of polymer—matallic powders mixtures with 5 wt% composition: **a** PA2201 + Ag; **b** PA2201 + Cu(S); **c** PA2201 + Cu(D)

The presence of fillers in the mixtures was also confirmed by SEM. Imaging was carried out using the BSE (backscattered electrons) mode. Due to the large difference in material densities and the atomic weight, it can be observed that the copper and silver particles, compared to the polymer, are areas of much greater brightness as shown in Fig. 5. This phenomenon is dictated by the backscattering of more electrons by heavier atomic nuclei.

**Fig. 6:**
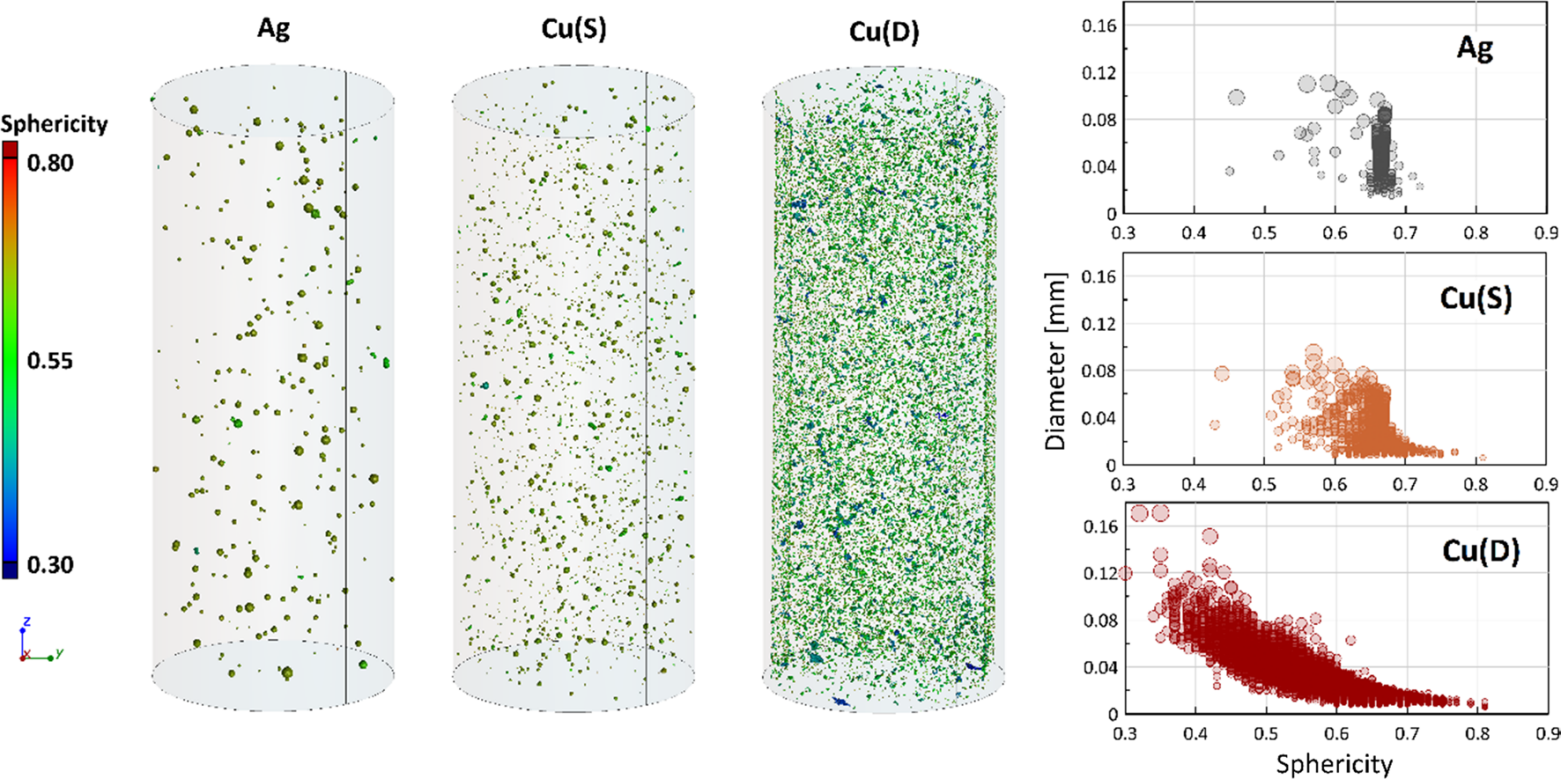
XCT images of Ag and Cu powders. The graphs show the relationship between the sphericity of the particles and their diameter

The quantitative evaluation of the shape of the powders was carried out using the XCT method. Figure 6 shows the analyzed powders with their size and sphericity marked in the form of a colour map. A sphericity factor of 1 corresponds to an ideal sphere, while the lower the sphericity value, the more the particle shape deviates from the ideal sphere. The most spherical shape is that of the Ag powder, where most of the particles are in a sphericity range from 0.65 to 0.70. Cu (S) powder is also characterized by high sphericity, where most of the grains are in the range of 0.60–0.75, but, in this case, the pores with the smallest diameters are characterized by the highest sphericity. Cu (D) powder is characterized by the lowest sphericity, where most of the particles are in the sphericity range from 0.4 to 0.7, reflecting the different shape of the dendritic powder.

**Fig. 7:**
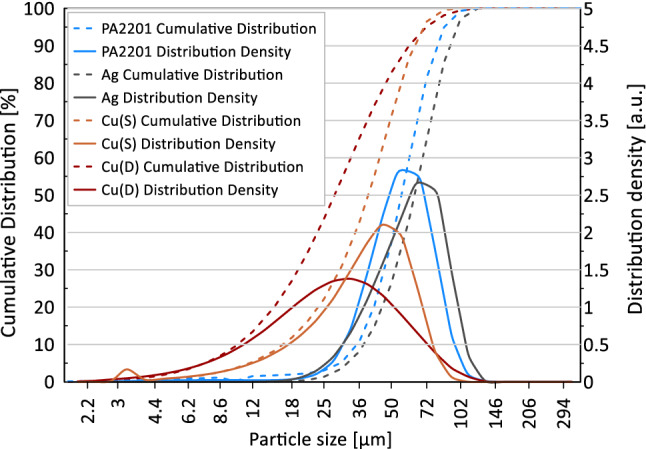
Particle size distribution of raw powders obtained by dry laser diffraction

The particle size distribution of base material and metallic filler measured using dry laser diffraction are presented in Fig. 7. PA2201 powder particles have a similar distribution as a widely used polyamide 12 (PA2200) [17], with a slightly lower amount (up to 2%) of particles below 20 µm. PA2201 particle distribution can be described as unimodal, symmetrical and narrow.

A similar distribution is represented by Ag powder, yet it is shifted to larger particle diameters, which is favourable for good flowability properties for polymer laser sintering. The spherical Cu powder shows bimodal and asymmetrical distribution. High content of small particles is confirmed by cumulative distribution curve, where up to 15% of particles below 20 µm are recorded. The second Cu powder, with a dendritic particle shape, has less favourable distribution, which can be described as wide and symmetrical, with an even higher content of small particles.

### Manufacturing process

**Fig. 8:**
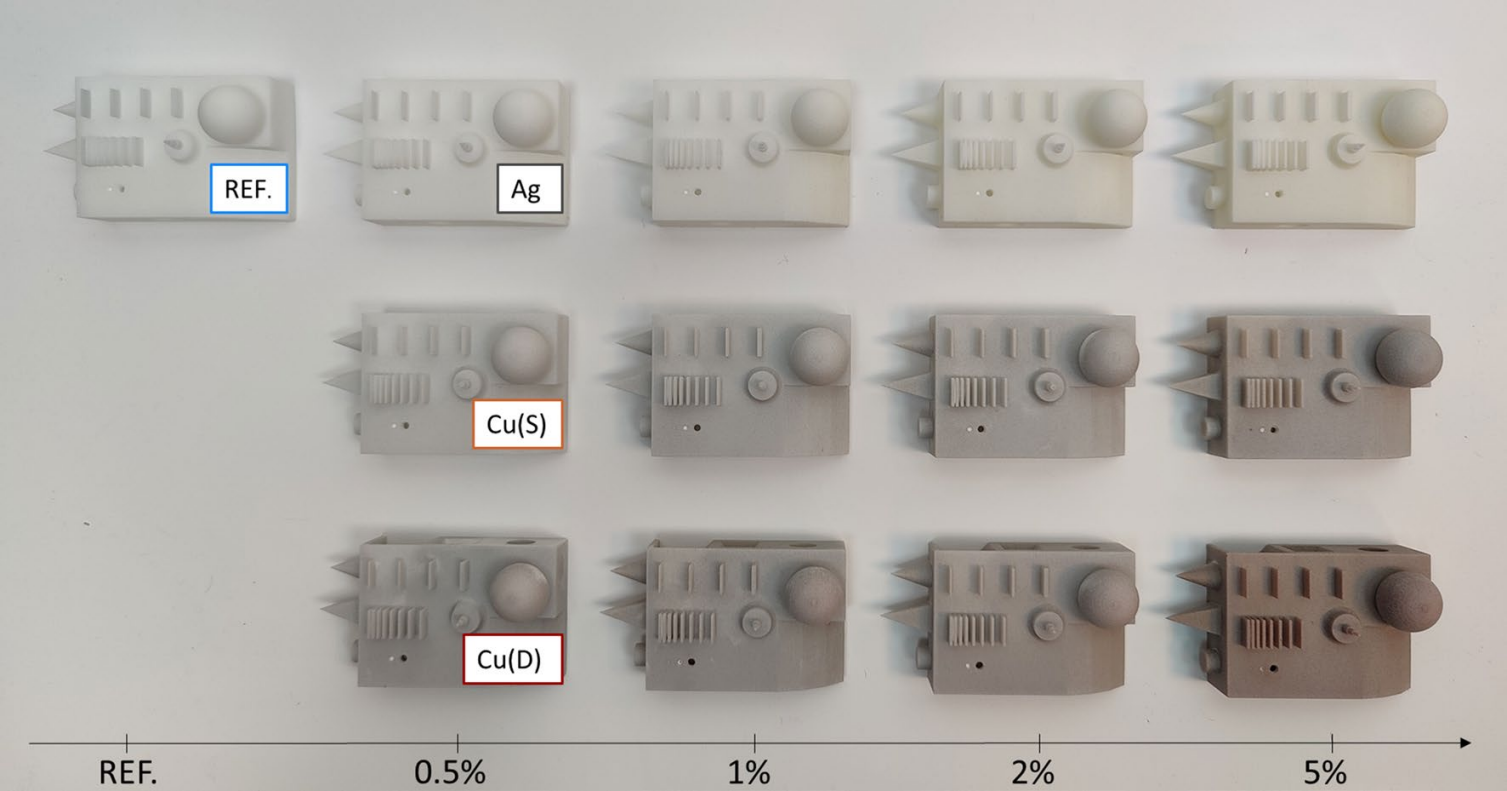
Benchmark geometry manufactured using base and mixture materials, first row—PA2201 + Ag, second row PA2201 + Cu (S), third row—PA2201 + Cu (D), filler content increase from left to right

Sample parts and specimens were manufactured in 13 different processes. An increase in the content of the filler did not require adjustments in processing temperatures. There were no visible disturbances or errors during the process. The produced models showed an increased colour change along with increasing filler content, as shown in Fig. 8. Each model was built correctly, with no visible differences, other than colour, to the reference material.

### Mechanical testing

**Fig. 9:**
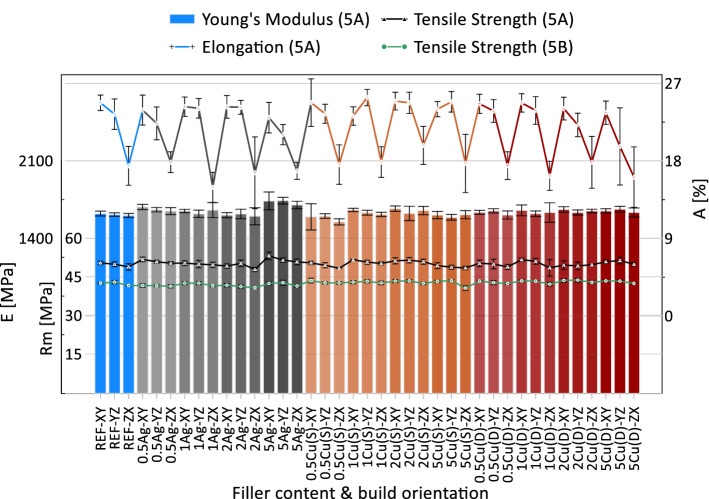
Mechanical properties of base and mixture materials presented for tensile specimens according to ISO-527-5A and ISO-527-5B standards

The presented mechanical properties are taken from a static tensile test of specimens prepared according to ISO-527-5A. Additionally, small samples (ISO-527-5B) with measured filler content allowed to determine its influence on tensile strength. The results of both static tensile tests, in relation to the designed filler content and build orientation, are shown in Fig. 9. The relatively low influence of increasing filler content on mechanical properties to the reference material can be observed, which is confirmed by both types of specimens. The tensile strength differs slightly and ranges from 47.82 to 53.14 MPa for 5A samples, and from 40.6 to 43.7 MPa for smaller ones (5B). The noticeable difference between tensile strength 5A and 5B in favour to larger samples is due to the lower ratio of the area scanned by hatching and contour style in a single layer [18], which shows that the manufacturer’s parameters are not optimized for small objects with a small layer area. In this paper, the samples built according to ISO-527-5B were intended to determine the achieved filler content and consider its influence on mechanical properties. The results (according to ISO-527-5A) do not deviate from the literature [19] and confirm that new polymer mixtures do not require parameters adjustments when considering mechanical properties. Anisotropy of mechanical properties is observed in the case elongation at break. Samples built in vertical (ZX) orientation are characterized by elongation at the break from 14.93 up to 19.80% where other two tested orientations differ from 19.68 to 25.36%. The highest influence is visible in the case of 5 wt% Ag as well as Cu(D), which resulted in an increase of Young’s Modulus and a decrease in elongation at the break in each build orientation. In case of a 5Ag series the elongation at break drop, simultaneous increase of Young’s Modulus confirms that 5 wt% content is a limit value where mechanical properties begin to change. The most visible negative effect of a filler is observed for samples built in *ZX* direction, whereas elongation at the break begin to change slightly at filler content of a 1 wt% and more. This behaviour is not desirable in case of considered applications. A further increase in weight content negatively affects the ductility of PA12, which was reported by Wolf in 10 wt% copper mixtures, where a significant drop in elongation at the break was observed [16].

### Fractography

**Fig. 10:**
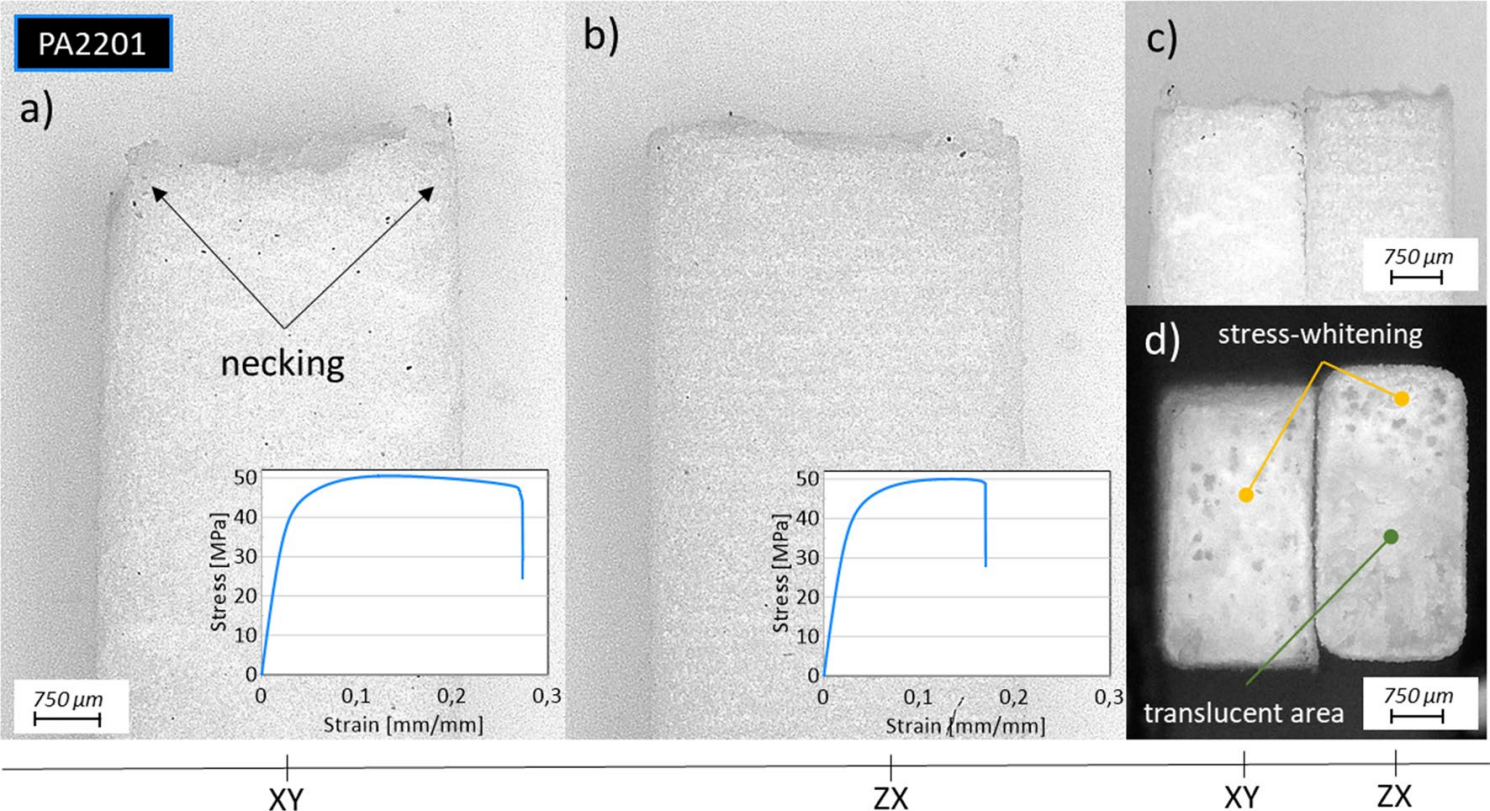
Microscopic images of tensile samples’ fracture surface manufactured with base material (PA2201) built in different orientation and presented from side: **a** *XY* from top view with representative stress–strain curve, **b** *ZX* from top view with representative stress–strain curve, **c** *XY* and *ZX* from side view, **d** *XY* and *ZX* fracture view

The damage behaviour is presented in Fig. 10 which reveals anisotropic nature of the deformation. The samples produced in the *XY* build orientation are characterized by plastic deformation before crack, which is confirmed by the necking visible in Fig. 10a, c. The different nature of the crack is visible in the case of samples manufactured in *ZX* build orientation. Vertically built specimens show no necking and break between successive layers, as presented in Fig. 10b, c. Fracture surfaces also show signs of the presence or absence of localized plastic deformation, which is revealed by whitening of the initially partially transparent polymer. The less translucent polymer in the fracture area corresponds to a higher plastic deformation occurring in the place of the crack, which in the literature is described as stress whitening. This effect can be noticed when comparing the *XY* and *ZX* samples presented in Fig. 10d.

The results obtained do not differ from those presented in the literature [20]. The whitening of the fracture surface of horizontally produced samples is caused by appearance of cavities [21]. The pores present in the PA12 samples produced with pLS are growing only if plastic deformation occurs, which was confirmed by Ziółkowski et al. [22].

**Fig. 11:**
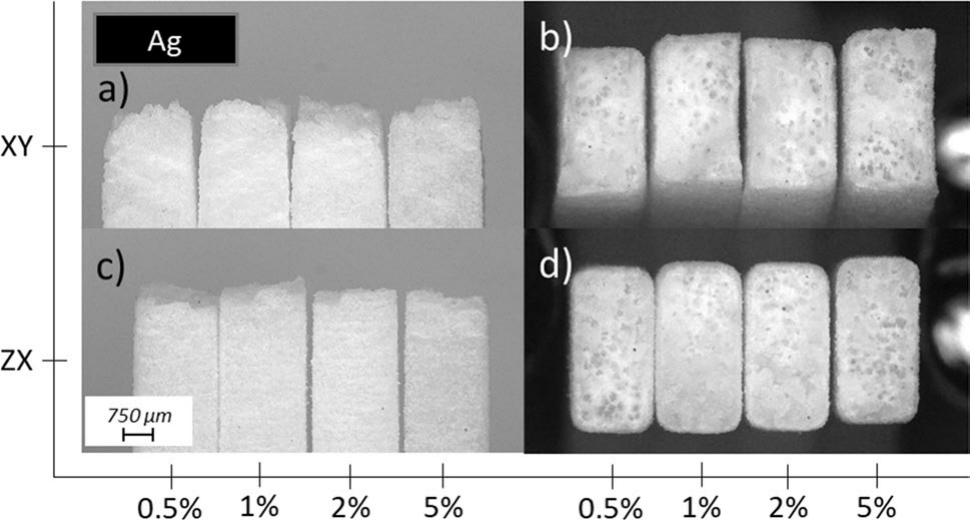
Microscopic images of tensile samples’ fracture surface manufactured with PA2201 + Ag and various filler content (0.5, 1.0, 2.0, 5.0 wt%) built in different orientation and presented from side: **a** *XY* from side view, **b** *XY* fracture view, **c** *ZX* from side view, **d** *ZX* fracture view

**Fig. 12:**
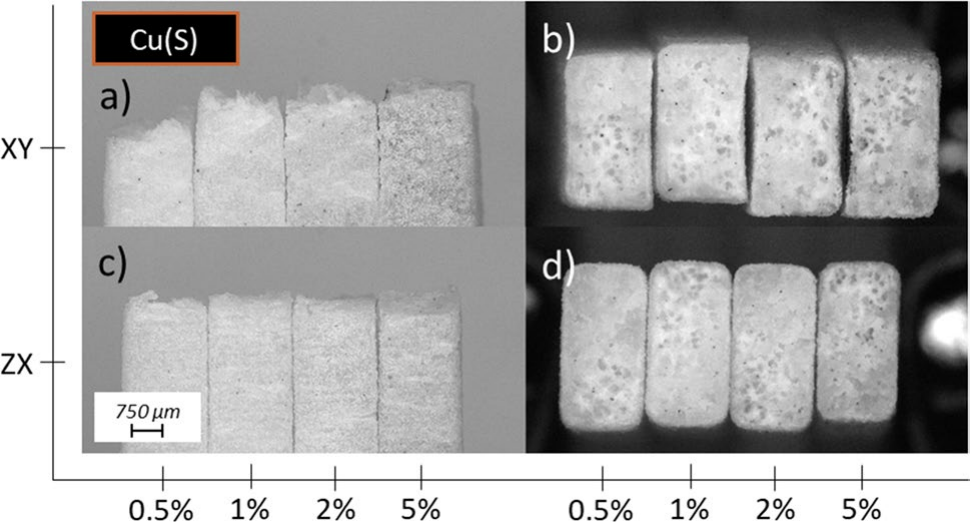
Microscopic images of tensile samples’ fracture surface manufactured with PA2201 + Cu(S) and various filler content (0.5, 1.0, 2.0, 5.0 wt%) built in different orientation and presented from side: **a** *XY* from side view, **b** *XY* fracture view, **c** *ZX* from side view, **d** *ZX* fracture view

**Fig. 13:**
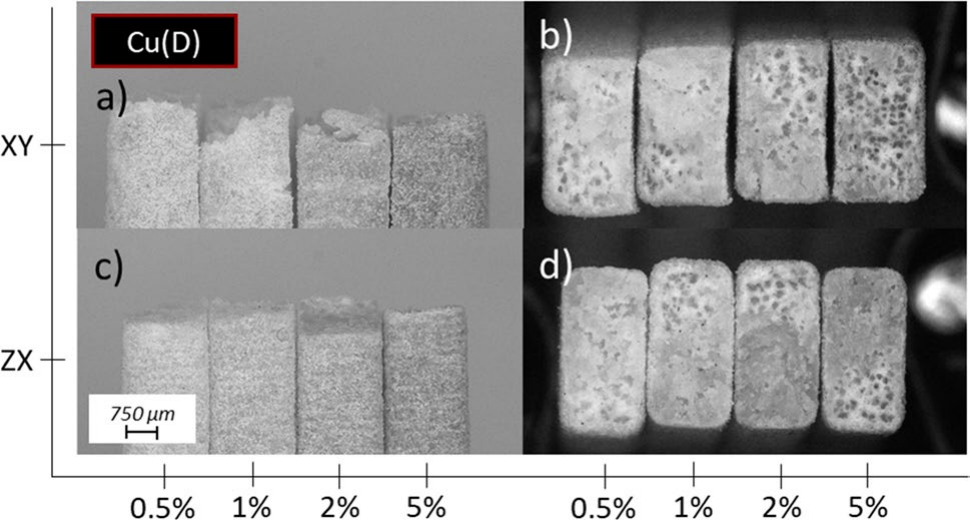
Microscopic images of tensile samples’ fracture surface manufactured with PA2201 + Cu(D) and various filler content (0.5, 1.0, 2.0, 5.0 wt%) built in different orientation and presented from side: **a** *XY* from side view, **b** *XY* fracture view, **c** *ZX* from side view, **d** *ZX* fracture view

The effects are also visible in the case of series with metallic fillers. Side and top view of the fracture for each representative sample can be found in Fig. 11a–d for Ag series, Fig. 12a–d for Cu(S) series and Fig. 13a–d for Cu(D) series. No significant changes in the fracture behaviour are observed for mixtures with low content materials, especially in the case of 0.5 and 1.0 wt%. The increase in filler content results in more brittle character of the fracture, which can be observed as a growing participation of translucent area in samples built in *XY* orientation. It is especially visible in case of Cu(D) series, where in Fig. 13b sample with 5.0 wt% has slightly white surface, along with smaller necking effect visible on Fig. 13a.

In the case of samples built in *ZX* direction, similar effect of stress whitening is observed, whereas with growing contribution of a translucent area its intensity increases, simultaneously confirming more brittle character of a damage, which can be clearly visible in Figs. 12d, 13d. Lack of necking and breaking between successive layers are visible in case of each representative of *ZX* sample, which is confirmed by microscopic images presented in Figs. 11c, 12c, 13c.

Moreover, the slight necking effect reported for the horizontally built samples fades away along with increasing content of a filler, which can be seen in Figs. 12a and 13a. The smallest change in fracture behaviour is observed for the series with Ag filler, where the necking it was not completely gone, as can be seen in Fig. 11a. This may be due to the size of the filler represented by the largest diameters. When comparing the shade of materials colour of both copper-filled series, as presented in Figs. 12a, c, 13a, c there are expected differences in the filler content in relation to other series with corresponding designed one.

Fracture analysis confirms the mechanisms occurring during the static tensile test and provides an excellent explanation of the phenomena accompanying the tensile of the material.

### NDT characterization with X-ray computed tomography (XCT)

**Fig. 14:**
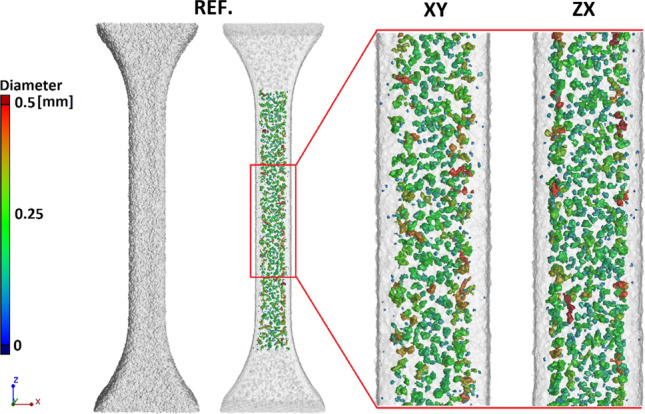
Example of the volumetric porosity determined for the reference sample (virgin PA2201), magnification for build directions in the *XY* and *ZX* plane

The XCT analysis was focused on detecting porosity within the processed material, both for virgin PA2201 samples and for samples with Ag and Cu fillers. In this case, 5 samples from each series were reconstructed with a resolution of 20 µm. Figure 14 shows an example of the porosity recorded with XCT in PA2201 samples. The porosity level is lower for XY orientation (vertical) and ranges from 2.7 to 3.5%, whereas ZX (horizontal) ranges from 1.5 to 2.5%. The porosity levels are typical as reported in the literature for pLS of PA12. Sitchel et al. verified the porosity level on six different pLS systems from different manufacturers and achieved results of around 3% [23], while Olejarczyk et al. testing manufacturer’s parameter sets reported porosity of around 5% for each build orientation [17]. Similar values were recorded by Dewulf et al., which ranged from 2.5% up to 4.7% [18].

**Fig. 15:**
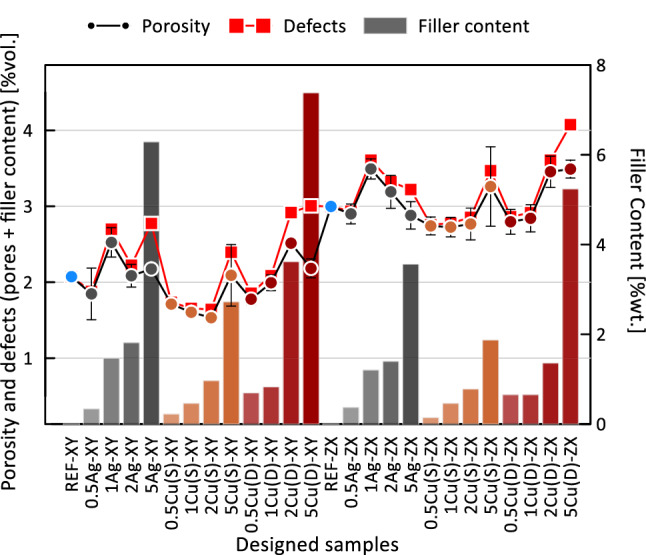
Volumetric porosity, volumetric defects (pores and filler content) and weight fraction of filler content in manufactured samples using base and mixture materials

No dependence of the amount of filler used on the porosity is observed. Moreover, the samples whose porosity exceeded 3% resulted in having a slightly lower elongation at the break, especially considering the higher filler content. This behaviour shows that both pores and metallic powder particles act like defects that are weakening the material. The overall volume percentage of defects considered as a sum of volume of pores, metallic particles and the mass percentage of additive filler is shown in Fig. 15.

**Fig. 16:**
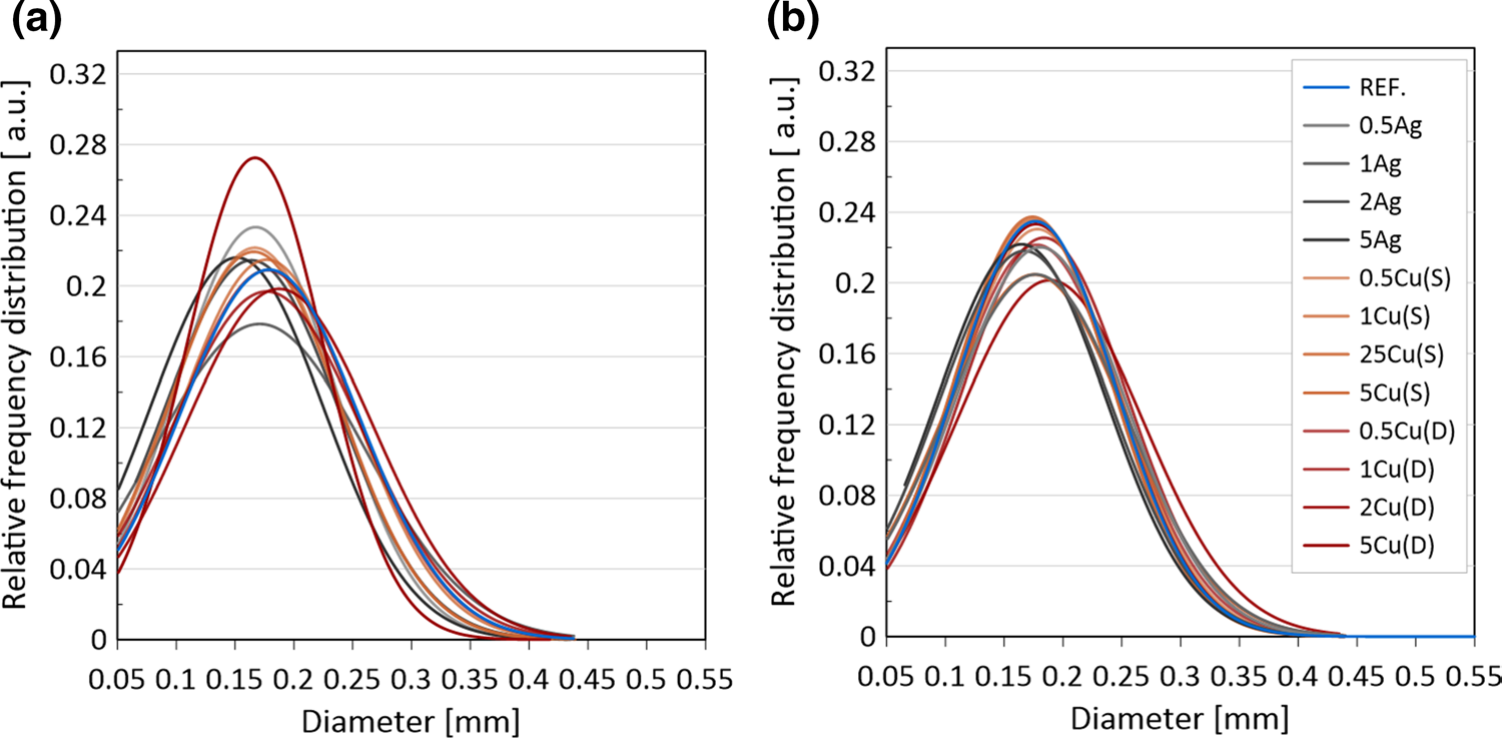
Pore size distribution of samples produced using base and mixture materials in build orientation: **a** *XY* and **b** *ZX*

The pore size distribution was determined and presented as a relative frequency distribution in relation to the pore diameter in Fig. 16. The distribution curves are unimodal, narrow and symmetrical. Higher repeatability is observed for the *XY* build orientation, and most of the mixture materials had a similar distribution or larger share of bigger pores than the reference sample.

At the same time, most samples with fillers and a build in the *ZX* direction had more smaller pores than the reference sample without fillers. Small visible changes in the porosity level of the samples, depending on the type and content of the metallic fillers differences, may be caused by process variability itself. The series which stands out is the sample built in *XY* using dendritic Cu at the 5 wt% blend. It showed significant increase in the content of pores between 150 µm and 200 µm. Additionally worth noticing is the effect which can be observed in the series built with an Ag filler and both build orientations. As the silver content increases, the share of smaller pores increases. In the case of other spherical filler- Cu(S), such a trend is only visible for samples at 5 wt%.

**Fig. 17:**
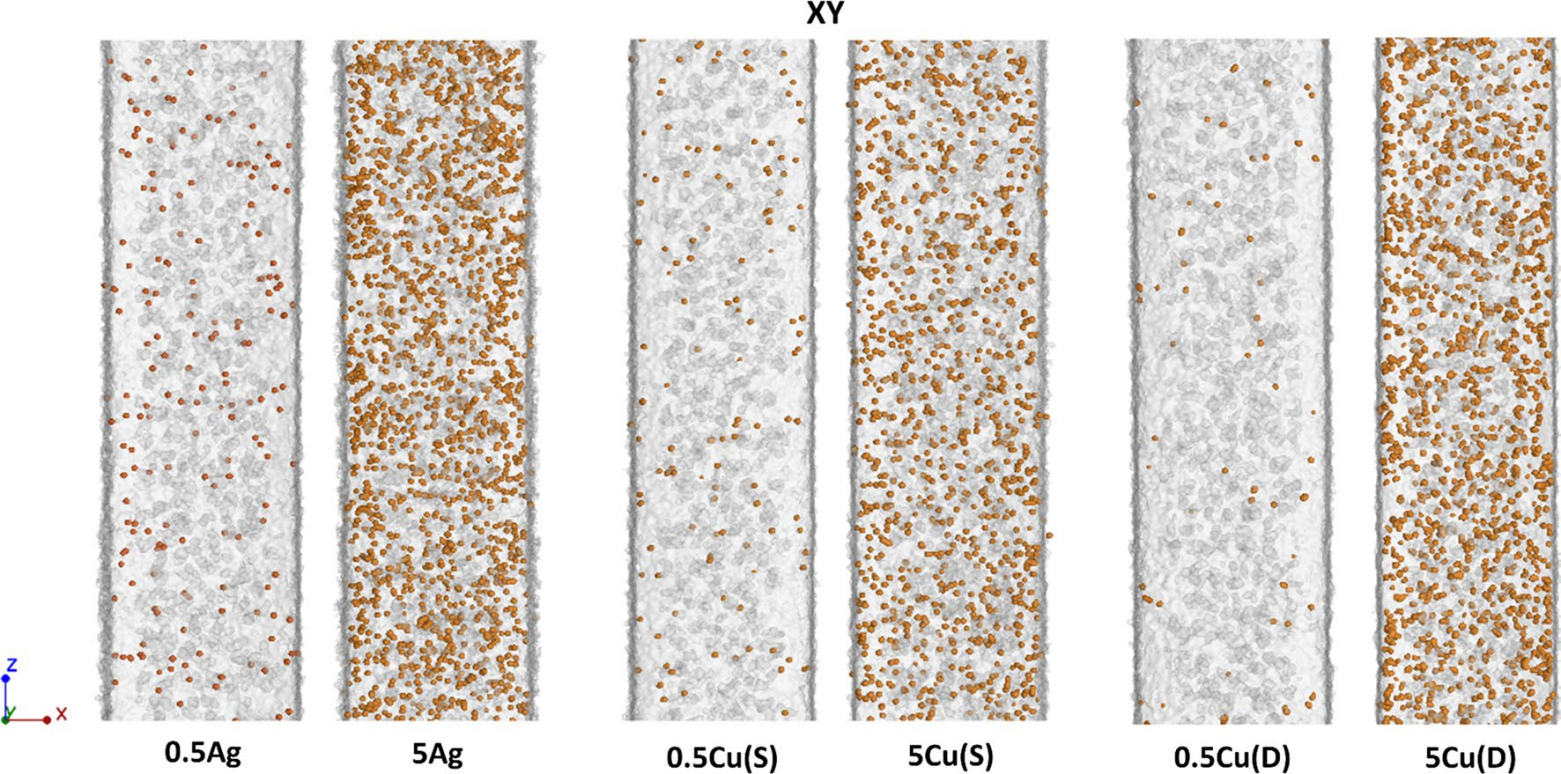
Filler content in samples built in *XY* direction, magnification for maximum and minimum values of fillers

Changes in the filler content for the minimum (0.5 wt%) and maximum (5 wt%) values of Ag, Cu(S) and Cu(D) fillers for samples built in the *XY* plane are shown in the Fig. 17. The 0.5Cu(D) samples have the lowest number of grains in the case of the minimum filler content. However, the grains observed here have the largest volume and weight. In the case of maximum filler content, the smallest amount of grain is registered in the 5Cu(S) sample, confirming the tendency presented in Fig. 8.

**Fig. 18:**
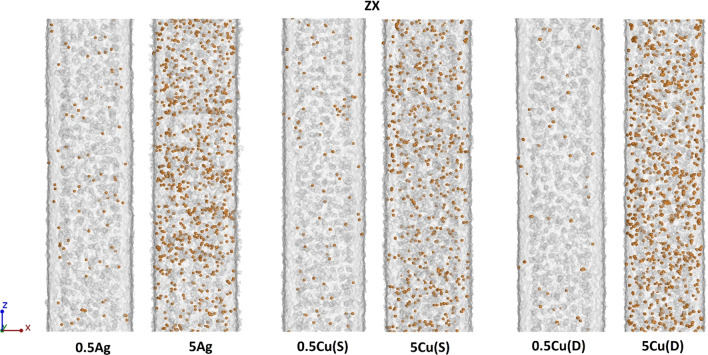
Filler content in samples built in *ZX* direction, magnification for maximum and minimum values of fillers

For the *ZX* build direction, a smaller number of the filler grains was registered than for the *XY* build direction, which is clearly visible when comparing 5 wt% filler content (Fig. 18).

**Fig. 19:**
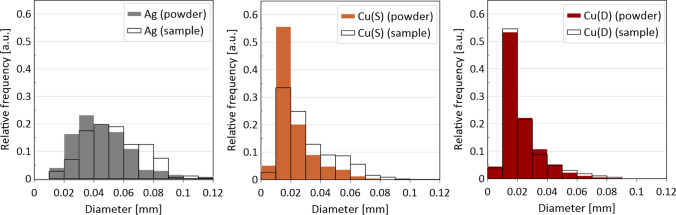
Relative frequency of metallic particle size distribution in powder mixture and manufactured samples

A more accurate evaluation of the nature of the powder grain distribution and their size was carried out thanks to the XCT reconstruction with a resolution of 2.9 µm. The measured filler content shows differences compared to the designed one. In most cases, for the *ZX* orientation, it proves to be lower than aimed. The small single layer area, which is present in the tensile specimen *ZX* build, is suspected to be responsible for the additive content decrease. The highest decrease of filler content was detected in the spherical copper samples series, which is also visible in the particle size distribution of metallic particles in the powder mixture and manufactured samples. This is also confirmed by elongation at the break which did not start changing at filler 1 wt% Cu(S) filler content. The relative frequency of fillers depending on their state before and after processing is presented in Fig. 19. Spherical fillers (Ag and Cu(S)) show a tendency to lose smaller particles (< 20 µm) during the manufacturing process. Fine powder content reduction is a behaviour known also from Laser Powder Bed Fusion of metals (LB-PBF/M) [24], which is explained by deposition on many surfaces in the machine chamber. In the case of mixture powders, the cause may be also sought in small particles sedimentation; thus the powder is in continuous movement during the delivery and application on work surfaces. The good flowability properties of spherical shape particles, especially for a smaller particle, favour such a behaviour.

To quantify the homogeneity of the filler distribution in the sample’s linear fit function to accumulated number of particles depending on a single slice (0.1 mm thickness) in a specific direction was determined. A perfectly distributed filler would be represented as fit factor *R* = 1 and the angle of the curve, *α* = 45°. The results obtained after the data analysis are presented in Table 2.

**Table 2:** Linear regression fit factor and slope of the fitting curve of metallic powder particle distribution

	Measurement direction													
	*X*	*Y*	*Z*	*X*	*Y*	*Z*	*X*	*Y*	*Z*	*X*	*Y*	*Z*		
	0.5Ag			1Ag			2Ag			5Ag				Build orientation
*R*	0.9946	0.9978	0.9955	0.9996	0.9967	0.9990	0.9989	0.9949	0.9996	0.9990	0.9992	0.9995	*XY*	
*α*	55.68	54.82	55.74	60.64	57.71	58.52	58.38	55.05	57.17	58.03	61.26	57.53		
	0.5Ag			1Ag			2Ag			5Ag				
*R*	0.9938	0.9779	0.9955	0.9983	0.9963	0.9974	0.9978	0.9977	0.9986	0.9989	0.9958	0.9941	*ZX*	
*α*	60.39	66.58	54.81	59.69	62.06	55.21	62.80	63.97	58.15	62.01	66.40	54.06		
	0.5Cu(S)			1Cu(S)			2Cu(S)			5Cu(S)				
*R*	0.9994	0.9982	0.9981	0.9989	0.9994	0.9997	0.9997	0.9997	0.9994	0.9994	0.9982	0.9981	*XY*	
*α*	58.60	61.70	59.89	56.16	57.92	58.07	57.33	59.52	58.58	58.60	61.70	59.89		
	0.5Cu(S)			1Cu(S)			2Cu(S)			5Cu(S)				
*R*	0.9999	0.9988	0.9989	0.9991	0.9981	0.9995	0.9997	0.9986	0.9992	0.9999	0.9988	0.9989	*ZX*	
*α*	57.49	57.26	58.39	59.11	61.04	58.05	56.24	61.03	59.16	57.49	57.26	58.39		
	0.5Cu(D)			1Cu(D)			2Cu(D)			5Cu(D)				
*R*	0.9998	0.9997	0.9985	0.9999	0.9991	0.9995	0.9999	0.9999	0.9991	0.9999	0.9996	0.9970	*XY*	
*α*	57.04	58.26	60.52	58.42	60.39	59.09	60.52	57.94	59.40	58.98	60.93	58.94		
	0.5Cu(D)			1Cu(D)			2Cu(D)			5Cu(D)				
*R*	0.9998	0.9990	0.9991	0.9999	0.9995	0.9992	0.9999	0.9987	0.9998	0.9992	0.9988	0.9989	*ZX*	
*α*	58.57	57.82	60.67	60.07	59.50	60.55	59.26	60.12	58.43	63.25	60.99	59.18		

**Fig. 20:**
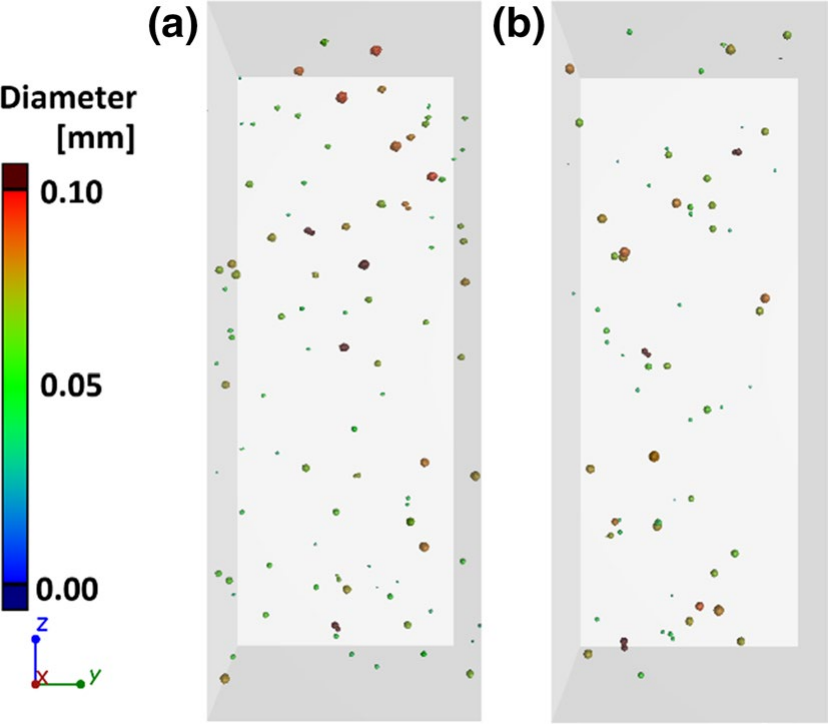
Different homogeneity of the powder grains distribution in the sample **a** 0.5Ag-*XY*—average values for combined *X*, *Y*, *Z* direction o $\overline{R }=\mathrm{0,9960}$, $\overline{\alpha }=\mathrm{55,41}$; **b** 0.5Ag-ZX—$\overline{R }=\mathrm{0,9891}$, $\overline{\alpha }=\mathrm{60,59}$

The compared samples have similar *R* and *α* values for the *Z* direction, but significantly differ in the case of the *Y* direction, being at the same time the best and the worst results achieved. Overall, the obtained results suggest that metal particle distribution can be described as good. No correlations between the type of filler or its content are visible. When considering direction as well as filler content, the samples built in an XY orientation tend to have smaller differences between each other. An example of samples with significant differences for parameters α and R is presented in Fig. 20.

## Conclusions

An investigation on the influence of bioactive metal filler microstructural homogeneity and mechanical properties of pLS processed PA12 was carried out. The evaluation included a base material (PA2201) and its 12 mixtures with 3 types of metallic fillers with a wt% of 0.5, 1.0, 2.0 and 5.0 respectively. Both the powder materials and the samples produced were analyzed focusing on powder morphology, mechanical properties and microstructural homogeneity. The key conclusions are:

1. The filler content up to a 5 wt% ratio does not influence mechanical properties significantly, including microstructural homogeneity, such as porosity and its size and distribution. The investigated material mixtures did not require process parameter adjustments. The 5 wt% ratio is a limit value where mechanical properties, especially elongation at break, start to change, and the negative effect of a filler is visible.
2. The behaviour of a damage is also affected by the filler content increase, whereas for the limit value of 5 wt% a smaller necking effect is observed for the *XY* direction along with less stress whitening for both build orientations.
3. Both pores and metallic inclusions act as defects, where a vol% of 3 is proven to be the limit value for which mechanical properties begin to change, especially when considering one of the parameters most sensitive to error—elongation at the break.
4. Powder mixtures with a low volumetric ratio of filler (all considered series) allows to manufacture samples with fair or good distribution of metallic filler. The small surface area of the single layer results in filler content reduction, as seen in samples 5B built in *ZX* orientation.

It is also worth emphasising:

1. A decrease of fine particle fraction (< 20 µm) in spherical or near-spherical powders during the process is observed, due to the following: deposition on machine surfaces and particle sedimentation during the manufacturing process (powder delivery and application).
2. Processing powder mixtures with dendritic shape fillers via pLS proved not to change particle size distribution, even of fine particles, which is favourable behaviour that can influence the least designed and actual filler content. These types of mixtures should be prepared with caution. Non-spherical powders tend to have worse flowability properties [25], which can also influence overall mixture flowability and lead to an increase of porosity and maximum pore size [26].
3. The increased content of spherical Ag causes pore fragmentation, represented in a higher share of smaller pores in pore size distribution.
4. By using the appropriate measurement parameters, XCT can be successfully used to evaluate filler morphology (e.g., metal) in which density differs significantly from the matrix material (e.g., polymer). Analysis can be done with feedstock material (powder mixture) as well as previously processed polymer blends.

For further investigation, which ultimately will allow the selection of bioactive material designed for pLS, microbiological testing is planned. New material biocompatibility, as well as its antimicrobial properties in relation to filler content, should be taken into consideration. Moreover, the flowability of powder blends in static and dynamic conditions should be taken into consideration since homogenous powder application is one of the key factors in achieving parts with uniform density with a small porosity level [26].
